# Two-step magnetic bead-based (2MBB) techniques for immunocapture of extracellular vesicles and quantification of microRNAs for cardiovascular diseases: A pilot study

**DOI:** 10.1371/journal.pone.0229610

**Published:** 2020-02-26

**Authors:** Shi Chen, Shu-Chu Shiesh, Gwo-Bin Lee, Chihchen Chen

**Affiliations:** 1 Institution of NanoEngineering and MicroSystems, National Tsing Hua University, Hsinchu, Taiwan; 2 Department of Medical Laboratory Science and Biotechnology, National Cheng Kung University, Tainan City, Taiwan; 3 Department of Power Mechanical Engineering, National Tsing Hua University, Hsinchu, Taiwan; 4 Institution of Biomedical Engineering, National Tsing Hua University, Hsinchu, Taiwan; University of California, Merced, UNITED STATES

## Abstract

Extracellular vesicles (EVs) have attracted increasing attention because of their potential roles in various biological processes and medical applications. However, isolation of EVs is technically challenging mainly due to their small and heterogeneous size and contaminants that are often co-isolated. We have thus designed a two-step magnetic bead-based (2MBB) method for isolation a subset of EVs as well as their microRNAs from samples of a limited amount. The process involves utilizing magnetic beads coated with capture molecules that recognize EV surface markers, such as CD63. Captured EVs could be eluted from beads or lyzed directly for subsequent analysis. In this study, we used a second set of magnetic beads coated with complementary oligonucleotides to isolate EV-associated microRNAs (EV-miRNAs). The efficiencies of 2MBB processes were assessed by reverse transcription-polymerase chain reaction (RT-PCR) with spiked-in exogenous cel-miR-238 molecules. Experimental results demonstrated the high efficiency in EV enrichment (74 ± 7%, *n* = 4) and miRNA extraction (91 ± 4%, *n* = 4). Transmission electron micrographs (TEM) and nanoparticle tracking analysis (NTA) show that captured EVs enriched by 2MBB method could be released and achieved a higher purity than the differential ultracentrifugation (DUC) method (*p* < 0.001, *n* = 3). As a pilot study, EV-miR126-3p and total circulating cell-free miR126-3p (cf-miR126-3p) in eight clinical plasma samples were measured and compared with the level of protein markers. Compared to cf-miR126-3p, a significant increase in correlations between EV-miR126-3p and cardiac troponin I (cTnI) and N-terminal propeptide of B-type natriuretic peptide (NT-proBNP) was detected. Furthermore, EV-miR126-3p levels in plasma samples from healthy volunteers (*n* = 18) and high-risk cardiovascular disease (CVD) patients (*n* = 10) were significantly different (*p* = 0.006), suggesting EV-miR126 may be a potential biomarker for cardiovascular diseases. 2MBB technique is easy, versatile, and provides an efficient means for enriching EVs and EV-associated nucleic acid molecules.

## Introduction

Many cell types release membrane-enclosed particles, known as extracellular vesicles (EVs), into the extracellular space as a means to transmit signaling and genetic information [1–3]. Based on their biogenesis pathways, EVs are typically categorized into three main subgroups: exosomes, microvesicles, and apoptotic bodies [4]. However, it remains technically challenging to isolate EVs into a homogeneous subgroup and hence we use either EV as the generic term or terms for EV subtypes reflecting their physical characteristics, such as small EVs, or biochemical composition, such as CD63-positive EVs, following the ISEV guidelines (MISEV2018) [5]. EVs can be obtained from various body fluids [6, 7] and carry cargos, including unique and selected subsets of proteins and nucleic acids from the parental cells [8, 9]. As a result, there are growing interests in EV biology and clinical potentials [10–12]. However, the progress in the field is impeded by the heterogeneity of EVs and contaminants that are often co-isolated due to overlapped physical and chemical properties [13, 14]. There is a need for the improvement of methodologies that can consistently generate pure and intact EVs to provide reproducibility within and among laboratories [15].

A variety of techniques have been used for the isolation of EVs and the extraction of EV-associated molecules [16]. However, a standardized, accurate and clinically-valid method is yet to be developed [5]. The differential ultracentrifugation (DUC)-based technique, requiring minimal reagents and sample pretreatments, is by far the most widely used method to isolate EVs from biological fluids [5]. However, it requires expensive ultracentrifugation equipment and a trade-off between yield and purity [17]. In addition, the high acceleration associated with the ultracentrifugation induces the degradation of EVs and the co-precipitation of protein aggregates which may affect the downstream analysis [18]. Other commonly used EV isolation methods including ultrafiltration and kit-based precipitation often yield more EVs but substantial contaminants than DUC [19].

**Fig 1:**
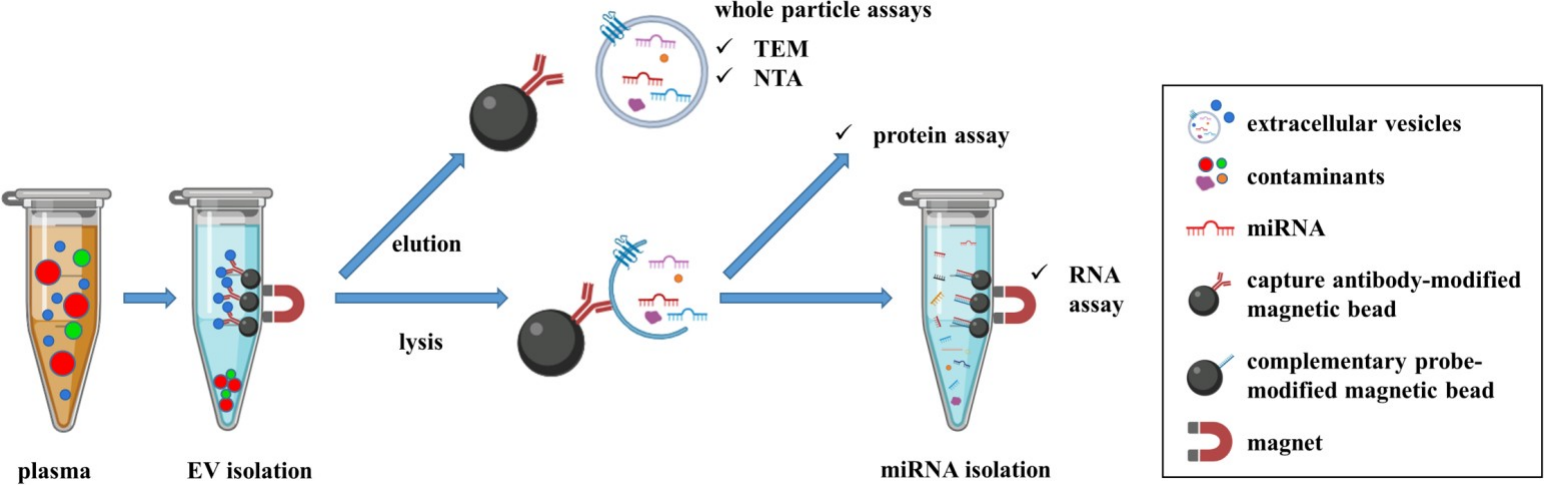
The schematic diagram of the experimental procedure. EVs in human plasma samples were first isolated using anti-CD63 antibody-coated magnetic beads. Captured EVs were then eluted for whole particle assays, such as transmission electron microscopy and nanoparticle tracking analysis. In addition, captured EVs were lysed for studying their molecular contents, including proteins and RNAs. EV-miRNAs were extracted using oligonucleotide-conjugated magnetic beads and subjected to subsequent analysis.

In order to increase the yield and purity of enriched EVs, we built on prior reported immune-affinity based techniques [14, 20, 21] and developed a two-step magnetic bead-based (2MBB) approach to recover EVs and EV-associated miRNAs. Micro-magnetic beads coated with capture molecules against EV-specific surface markers, such as CD63, could improve the enrichment process due to the high surface-to-volume ratio of beads. In this study, we aimed to determine the clinical performance of 2MBB. EVs were enriched from a small volume of plasma samples. Quantitative reverse transcription-PCR (RT-qPCR), protein assay, nanoparticle tracking analysis (NTA), and transmission electron micrographs (TEM) were used to assess and verify the efficiency, purity, amount, and morphology of enriched EVs. Fig 1 shows the schematic illustration of 2MBB and downstream analyses conducted in this study.

## Materials and methods

### Materials

Vacutainer tubes containing acid citrate dextrose (ACD) anticoagulant, anti-CD63 antibody-conjugated magnetic beads (catalog no.: 10606D), magnetic rack (catalog no.: 12321D), TaqMan^®^ miRNA ABC purification kit–Human Panel A, TaqMan^®^ microRNA reverse-transcription kit, MicroBCA^™^ Protein Assay Kit, phosphate-buffered saline (PBS), RIPA buffer, and proteinase inhibitor were obtained from Thermo Fisher Scientific (Waltham, MA, USA). Cel-miR-238-3p and miR-126-3p were purchased from Genomics (New Taipei City, Taiwan). Lyophilized bovine serum albumin (BSA) and prostaglandin E1 (PGE1) were obtained from Sigma-Aldrich (St. Louis, MO, USA). Iodixanol (OptiPrep) was obtained from Axis-Shield (Dundee, Scotland, UK). Sodium cacodylate buffer and 300-mesh Formvar/carbon-coated grids were obtained from Electron Microscopy Sciences (Hatfield, PA, USA).

### Collection of plasma samples

Blood samples were collected and provided by National Cheng Kung University Hospital (NCKUH, Tainan, Taiwan), under the approval of the Institutional Review Board (IRB) of NCKUH (IRB #B-ER-104-116). The need for consent was waived by the ethics committee. Fasting peripheral venous blood was collected using a 22-gauge needle. The first 3 mL of blood was discarded, and then blood samples were collected by venipuncture into vacutainer tubes. Blood samples were diluted with an equal volume of HEPES-NaCl buffer (10 mM HEPES, 0.85% NaCl, pH 7.4), and added with a final concentration of 10% ACD and 100 ng/mL PGE1 to prevent centrifuge-induced platelet activation. Six milliliters of diluted blood was layered on top of 3 mL of OptiPrep barrier solution with 1.077 g/mL in density and centrifuged at 700 × g for 20 min. The supernatant was subjected subsequent centrifugation steps of 300 × g for 10 min, 2,500 × g for 10 min twice to obtain platelet-poor plasma (PPP). All centrifugation steps were conducted at room temperature. These samples were kept in liquid nitrogen until use.

### EV enrichment using differential ultracentrifugation (DUC)

Plasma or serum sample was subjected to subsequent centrifugation steps: 300 × g for 10 min and 2,500 × g for 10 min twice at room temperature, followed by 10,000 × g for 40 min at 4°C in a 221.12V03 rotor (Hermle, Germany), and 100,000 × g for 90 min at 4°C in a type 70 Ti rotor using Optima XE ultracentrifuge (Beckman Coulter, Brea, CA, USA). The supernatant was removed, and the pellet was resuspended in 100 μL of PBS.

### EV enrichment using magnetic beads (MB)

EVs were enriched using magnetic beads according to the manufacturer’s protocol. In brief, anti-CD63 antibody-conjugated magnetic beads (100 μL, bead diameter 4.5 μm, 1×107 beads/mL) were first washed with 500 μL of the isolation buffer (0.1% BSA in PBS), and concentrated by placing on the magnetic rack for 1 min. After the removal of the supernatant, 100 μL plasma sample was added and mixed on a thermomixer at 4°C for 18 hr. EVs captured on magnetic beads were concentrated on the magnetic rack for 1 min, and the supernatant was aspirated carefully without disturbing the magnetic beads and collected. After washing twice briefly with the isolation buffer, captured EVs could be either lyzed directly for studying their molecular contents or eluted for whole-particle assays. EVs bound to magnetic beads were eluted by incubation in 100 μL of PBS containing 1% formic acid for 10–30 min. Eluted EVs were subsequently prepared for TEM, or diluted 100 times in PBS for NTA analysis.

### Extraction of EV-miRNAs using magnetic beads

EV-miRNAs were extracted using TaqMan^®^ miRNA ABC purification kit containing Human Panel A magnetic beads according to the manufacturer’s protocol. In brief, EVs were lysed by adding 100 μL of ABC lysis buffer on ice. Synthetic exogenous cel-miR-238-3p molecules were spiked in the lysate to a final concentration of 100 fM for normalization purposes. Anti-miRNA magnetic beads (80 μL containing 8×10^7^ beads) were first concentrated by placing on the magnetic rack for 1 min and the supernatant was discarded. The beads and the lysate were mixed on a thermomixer at 30°C with shaking at 1,200 rpm for 40 min. Magnetic beads were washed with subsequent 100 μL proprietary ‘wash buffer 1’ and 100 μL proprietary ‘wash buffer 2’. EV-miRNAs were eluted by mixing with 100 μL elution buffer at 70°C, 1,200 rpm for 3 min. The sequences of relevant target miRNAs detected by the Human Panel A beads were listed in S1 Table.

### Reverse transcription

EV-miRNAs of interest were first converted to cDNA using TaqMan^®^ microRNA reverse-transcription (RT) kit and the associated mi-RNA-specific stem-loop primers according to the manufacturer’s protocol. In this study, miR-21-5p, miR-126-3p, as well as spike-in cel-miR-238-3p were evaluated. 5 μL of EV-miRNA extract was added to the reaction mix containing 0.15 μL of 100 mM dNTPs, 1 μL of multiple reverse transcriptases, 1.5 μL of 10× RT buffer, 0.19 μL of RNase inhibitor, 4.16 μL of nuclease-free water, 3 μL of 5× RT primer to obtain a final volume of 15 μL. RT reaction was performed at 16°C for 30 min, 42°C for 30 min, and 85°C for 5 min.

### Real-time quantitative PCR (RT-qPCR)

To amplify cDNAs, 1 μL of Taqman small RNA Assay and 10 μL of 2× Taqman Universal PCR Master Mix were mixed with 2 μL of the cDNA sample, and RNase-free water was added to make the final volume 20 μL. All RT-qPCRs were carried out on a Real-Time PCR System (StepOnePlus^™^, Thermo Fisher Scientific, Waltham, MA, USA) with the following protocol: 50°C for 2 min, 95°C for 10 min; followed by 40 cycles of 95°C for 15 s and 60°C for 60 s. Relative miRNA expression levels were determined using the comparative cycle threshold, or 2^-ΔΔ*CT*^ method using the spike-in exogenous cel-miR-238-3p as the reference.

### Transmission electron microscopy (TEM)

50 μL of EV preparation was first fixed in 50 μL of 4% glutaraldehyde in 0.1 M sodium cacodylate buffer, pH 7.4. 10 μL of EV preparation sample was deposited onto a 300-mesh Formvar/carbon-coated grid. Excess liquid was blotted from the side with a filter paper after 10 min. For negative staining EVs, the TEM grid was first transferred to a 10-μL drop of 1% phosphotungstic acid solution that had been neutralized to pH 7 by adding sodium hydroxide and then to a 10-μL drop of distilled deionized water. Excess liquid was blotted from the side with a filter paper 1 min after each of these steps. After drying at 37°C for 10 min, the TEM grid was ready for imaging EVs at 80–120 kV using a Hitachi H-7100 transmission electron microscope (Hitachi High-Technologies Corp., Tokyo, Japan).

### Nanoparticle tracking analysis (NTA)

Nanoparticle tracking analysis (NTA) was used to analyze the size and concentration of EVs. EV samples were diluted with 0.1 μm-filtered PBS to achieve a recommended measurement concentration between ~10^8^–10^9^ particles/mL. All NTA measurements were carried out in triplicate at a frame rate of 25 frames/s using a NanoSight NS300 (Malvern Panalytical Ltd., Malvern, Worcestershire, UK) with a 488-nm laser. The temperature was maintained at 25°C. The camera level was 14 with the detection threshold set to 5. Data were analyzed using NanoSight NTA software version 3.2.

### Total protein assay—MicroBCA

The total protein content of EVs was determined using the MicroBCA Protein Assay Kit according to the manufacturer’s protocol using BSA as a standard. EVs were first lysed in RIPA buffer containing 1% proteinase inhibitor on ice for 10 min. 150 μL of lysate was mixed with 150 μL of working reagent at 200 rpm for 30 s and incubated at 37% for 2 hours. The absorbance was measured at a wavelength of 562 nm using a plate reader (POLARstar Omega, BMG Labtech, Ortenberg, Germany).

### Statistical analysis

Experiment data expressed with mean ±standard error of the mean (SEM) were tested in three or more independent replicates. One-way analysis of variance (ANOVA) and Student’s *t*-test were executed using Microsoft Excel 2019. A *p*-value < 0.05 was considered statistically significant and indicated by an asterisk (*); a *p*-value < 0.01 was indicated by two asterisks (**).

## Results

### Nanoparticle levels following the MB enrichment and elution of EVs in plasma samples

**Fig 2:**
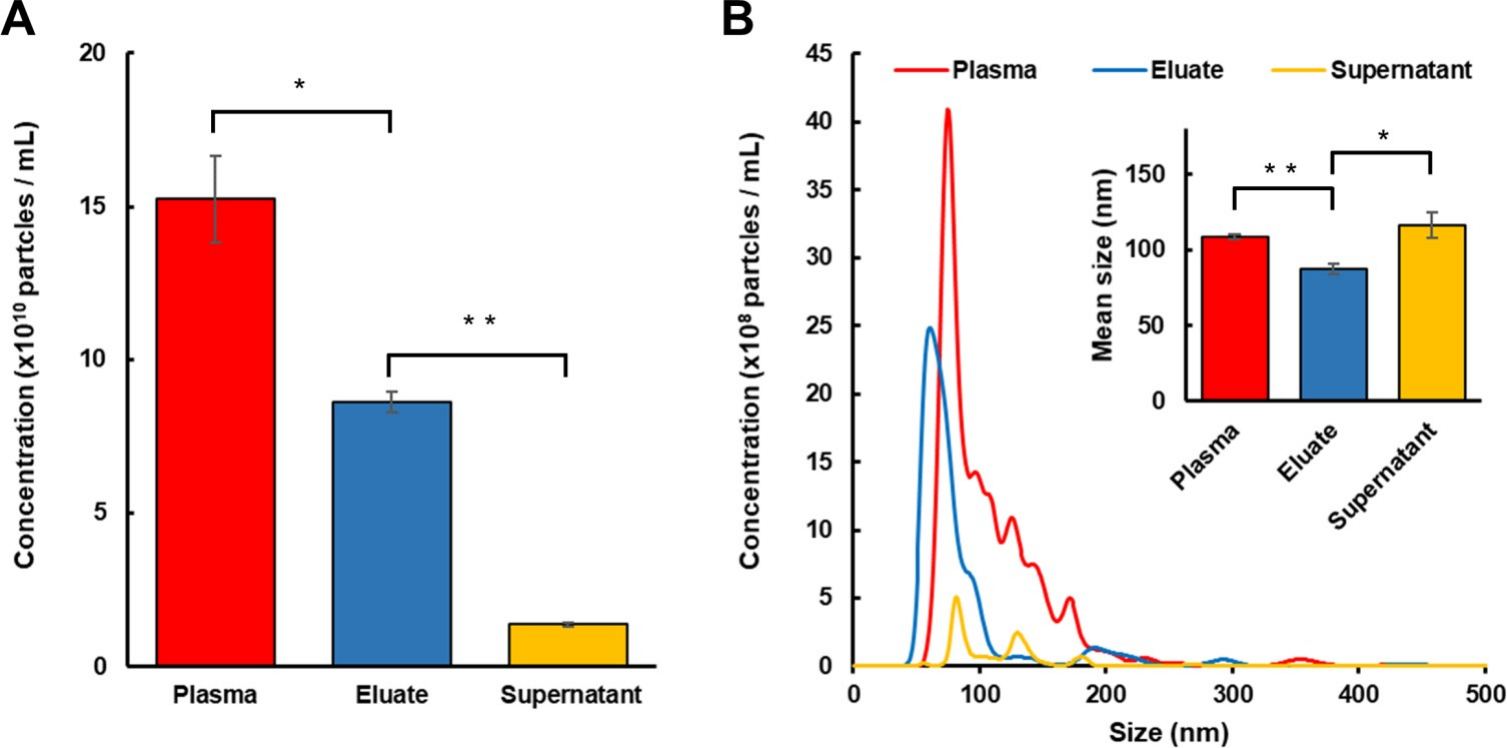
Nanoparticle analysis (NTA) of supernatants and eluates. NTA was utilized to determine the number (A) and size profile (B) of nanoparticles in the starting platelet-poor plasma (PPP), supernatant after EV enrichment, eluate after formic acid-mediated EV release from magnetic beads. Data are expressed as mean ± SEM (*n* = 3, ** *p* < 0.01, * *p* < 0.05, Student’s *t*-test).

The efficiency of enrichment and elution of plasma EVs using anti-CD63 magnetic beads was assessed by comparing the nanoparticle levels using NTA. Our results revealed that platelet-poor plasma (PPP) from the healthy donor contained 15.2 ± 1.42 × 10^10^ particles/mL and following mixing with anti-CD63 magnetic beads the number decreased to 1.4 ± 0.06 × 10^10^ particles/mL in the supernatant (Fig 2A, *n* = 3). The eluate contained 8.6 ± 0.34 × 10^10^ particles/mL, suggesting the overall recovery efficiency of total EVs calculated as the ratio of the number of particles in the eluate to that in PPP was about 57 ± 6%. Since CD63 is one of the classical markers for small EVs (sEV) [14], we further analyzed the concentrations of sEV (<100 nm) in different fractions (Table 1). NTA data indicated 86 ± 9% of sEV were recovered in the eluate.

**Table 1:** Enumerations and size profiling of particles using NTA. Small EVs (sEV) were defined as EVs smaller than 100 nm in diameter. Data are expressed as mean ± SEM from three measurements.

Source	Plasma	Eluate	Supernatant
**Mean diameter (nm)**	109.1 ± 1.6	87.4 ± 3.8	116.3 ± 8.5
**Median diameter (nm)**	91.9 ± 1.6	69.2 ± 1.3	105.8 ± 9.5
**Mode diameter (nm)**	74.7 ± 1.2	66.2 ± 4.1	81.9 ± 0.7
**Concentration (×10**^**10**^ **particles / mL)**	15.2 ± 1.4	8.6 ± 0.3	1.4 ± 0.1
**sEV Concentration (×10**^**10**^ **particles / mL)**	8.6 ± 0.8	7.4 ± 0.3	0.7 ± 0.03

The size distribution of nanoparticles in PPP appeared non-symmetric with a prominent peak at 74.7 ± 1.2 nm and several peaks at multiple diameters greater than 100 nm (Fig 2B, *n* = 3). In comparison, the size distribution of EVs eluted from magnetic beads was more symmetric, with relatively close median and mode diameters of 69.2 ± 1.3 nm and 66.2 ± 4.1 nm, respectively. Nanoparticles remained in the supernatant were generally larger, with a mean diameter of 116.3 ± 8.5 nm and a mode diameter of 81.9 ± 0.7 nm.

### TEM and protein levels of MB eluates and DUC concentrates

**Fig 3:**
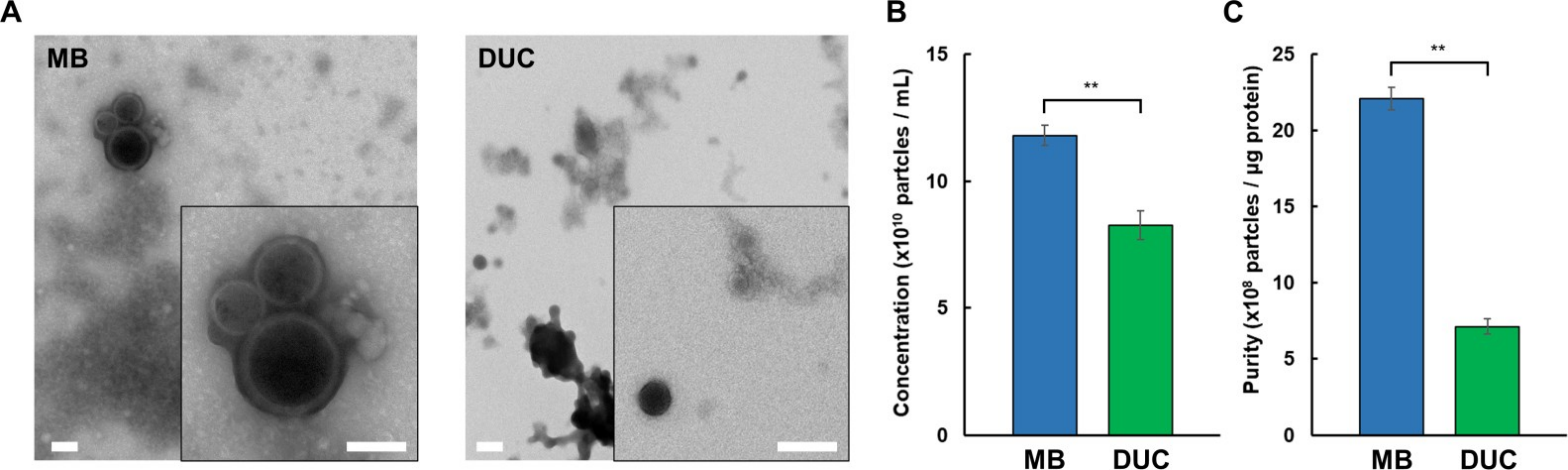
Nanoparticle and protein analysis of MB eluates and DUC concentrates. Transmission electron micrographs of negatively stained EVs isolated and eluted from magnetic beads (MB) or pelleted by differential ultracentrifugation (DUC) methods from PPP. Scale bars represent 100 nm. The double-layer character of EV membranes could be discerned at increased magnification (insets) (A). Enumeration of nanoparticles in MB eluate and DUC concentrate by NTA (B). Protein levels were quantified using MicroBCA assays. The ratio of nanoparticle concentration to μg protein was plotted as a relative measure of purity (C). Data are expressed as mean ± SEM from three measurements (** *p* < 0.01, Student’s *t*-test).

We compared the quantity and quality of EVs recovered from PPP by using magnetic beads (MB) and conventional differential ultracentrifuge (DUC). To this end, we first confirmed the presence of EVs by TEM (Fig 3A). The micrographs showed intact EVs containing lipid bilayers were present in both MB eluate and UC concentrate. Concerning EV recovery and purity, the evaluation as determined by NTA and MicroBCA revealed that MB recovered 43 ± 11% more particles (Fig 3B, *n* = 3) and 54 ± 1% fewer proteins compared to DUC, which resulted in a three-fold increase of purity as estimated by the ratio of nanoparticles counts to μg protein (Fig 3C, *n* = 3).

### miRNAs levels following the 2MBB concentration

**Fig 4:**
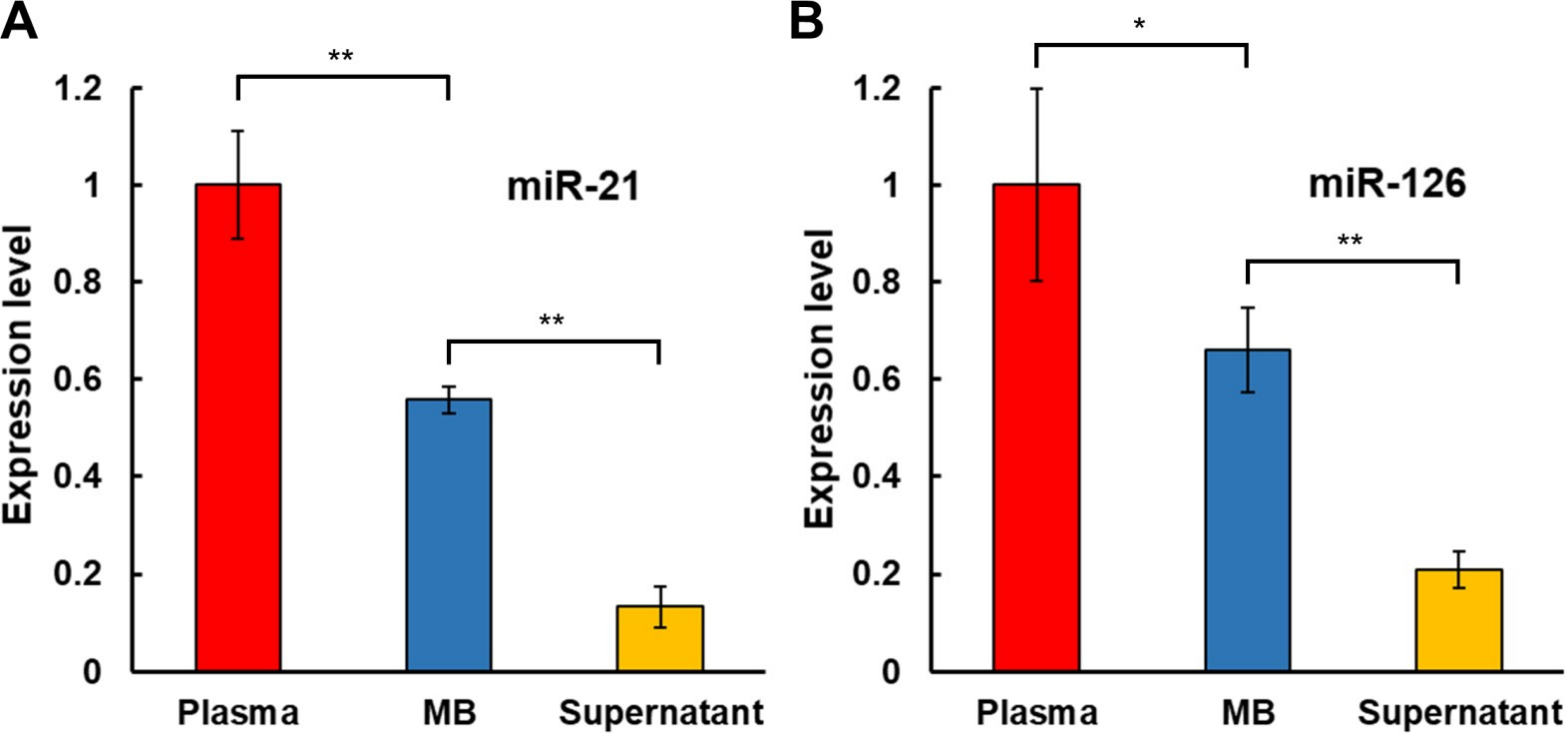
miRNA levels in plasma samples. Levels of miRNAs hsa-miR-21-5p (*n* = 4) (A) and hsa-miR-126-3p (*n* = 5) (B) extracted from platelet-poor plasma, EVs captured on magnetic beads (MB), and supernatant after MB concentration were measured relative to the spike-in exogenous cel-miR-238-3p by RT-qPCR. Data are expressed as mean ± SEM (** *p* < 0.01, * *p* < 0.05, Student’s *t* test).

We further assessed the amount of EVs isolated on anti-CD63 antibody-coated magnetic beads through measuring levels of miRNAs extracted from the starting plasma, EVs captured on magnetic beads (MB), and supernatant after MB concentration. Quantification of relative miRNA expression levels was determined using the comparative *C*_*T*_ method with the spike-in control of 100 fM of exogenous cel-miR-238-3p. Results showed that the EV capture rate, evaluated by the formulas below, was 56 ± 3% (Fig 4A, *n* = 4) and 66 ± 9% (Fig 4B, *n* = 5), which was comparable to the overall recovery efficiency determined by the enumeration of eluted EVs.

$$Capture\;rate=\frac{{\left[miRNA\right]}_{MB}}{{\left[miRNA\right]}_{plasma}}$$ (1)

### Correlations between miR-126-3p, cTnI and NT-proBNP levels in the plasma of healthy donors and CVD patients

**Fig 5:**
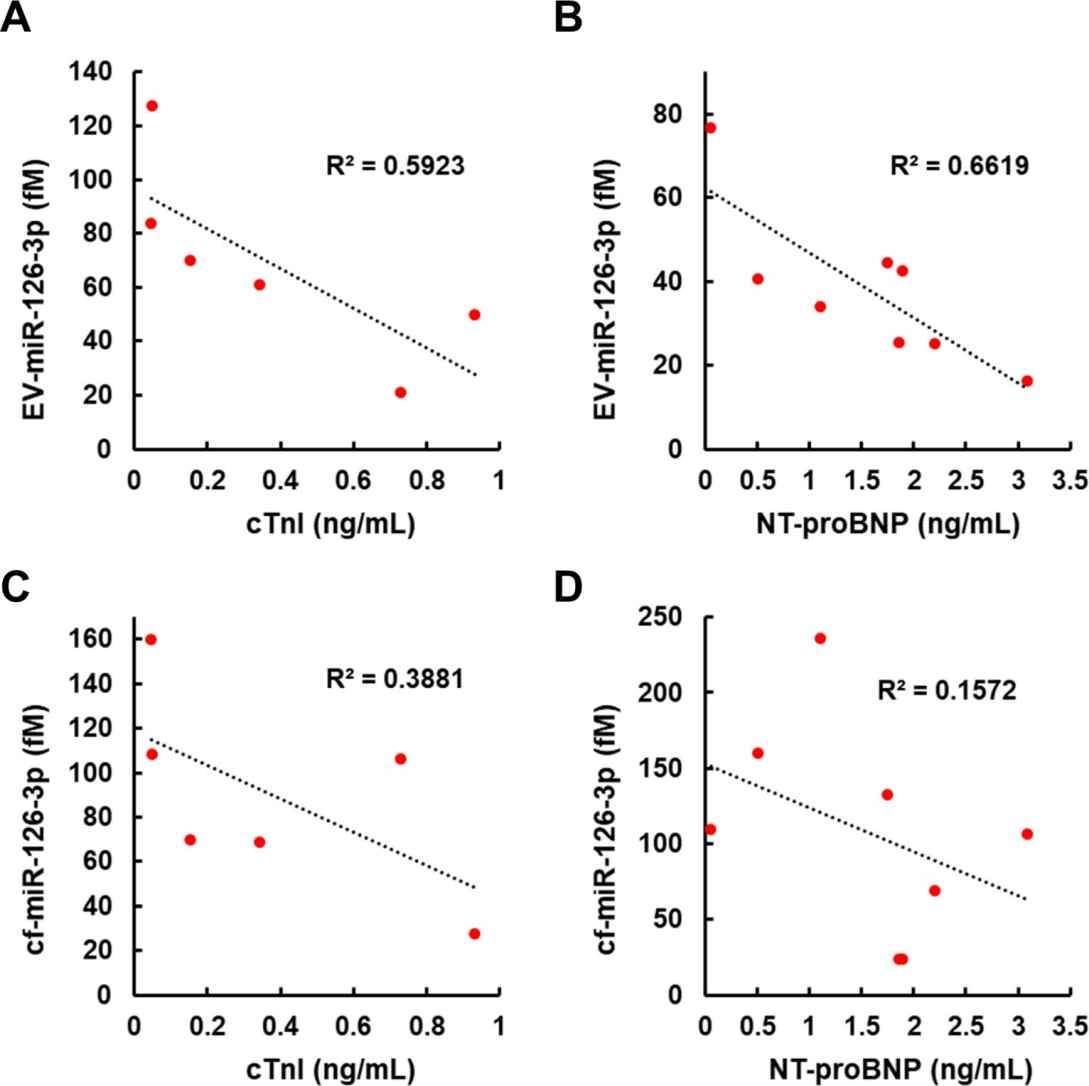
Levels of EV-miR-126-3p and cf-miR-126-3p were quantified and compared with the levels of conventional CVD biomarkers. Concentrations of cTnI and NT-proBNP were determined by enzyme-linked immunosorbent assays (ELISA) performed in NCKUH. EV-derived miR-126-3p showed negative correlations with cTnI (*n* = 6) (A) and NT-proBNP (*n* = 8) (B), while cell-free miR-126-3p showed weaker correlations with both protein biomarkers (C and D).

**Fig 6:**
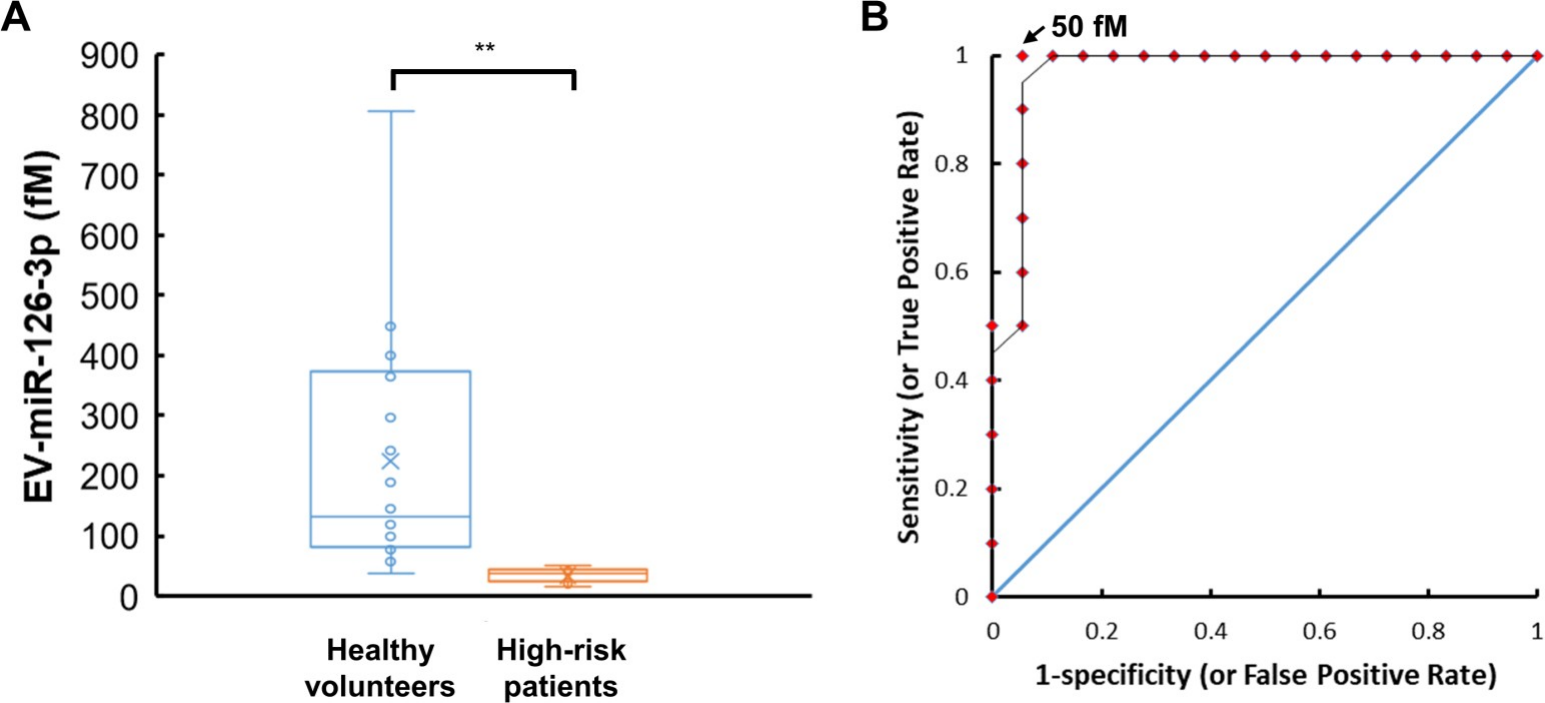
Levels of EV-miR-126-3p in plasma samples from healthy volunteers and high-risk CVD patients. A box-and-whisker chart shows measured EV-miR-126-3p concentrations in plasma samples from healthy volunteers (*n* = 18) and CVD patients with elevated levels of cTnI (> 0.5 ng/mL) or NT-proBNP (> 0.125 ng/mL) (*n* = 10) (A). The sensitivity and specificity were 100% and 94%, respectively, when the threshold EV-miR-126-3p concentration was 50 fM. The area under the receiver operating characteristic (ROC) curve was close to 1 and much greater than that of a randomly selected case shown as a straight line at a 45° angle (B).

We explored the capability of the 2MBB method to recover miR-126-3p molecules from EVs as well as directly from the plasma. EV-miR126-3p and total circulating cell-free miR126-3p (cf-miR126-3p) in clinical plasma samples were measured and compared with the level of widely-used CVD protein biomarkers, cTnI and NT-proBNP. Compared to cf-miR126-3p, an increase in correlations between EV-miR126-3p and cTnI, EV-miR126-3p and NT-proBNP was detected (Fig 5). We further compared EV-miR126-3p levels in plasma samples from healthy volunteers and high-risk CVD patients with elevated levels of cTnI (> 0.5 ng/mL [22]) or NT-proBNP (> 0.125 ng/mL [23]). The average EV-miR126-3p levels in these two groups were 224 ± 194 fM (*n* = 18) and 35 ± 11 fM (*n* = 10), respectively. One-way ANOVA analysis indicated a significant decrease in EV-miR126-3p levels in high-risk CVD patients (*p* = 0.006) (Fig 6A). A receiver operating characteristic (ROC) curve was used to assign a potential threshold concentration of EV-miR126-3p to separate these two groups (Fig 6B). A greater EV-miR126-3p level increased the sensitivity as fewer patient samples were excluded; however, the specificity would decrease as healthy volunteer samples might be included as well. The optimal threshold based on our small size of samples was 50 fM, which conferred a sensitivity of 1 and a specificity of 0.94, suggesting EV-miR126 may be a potential biomarker for cardiovascular diseases (CVDs).

## Discussion

EVs are membrane-bound particles that can be found in various biological fluids, such as blood, urine, cerebrospinal fluids, and breast milk. In order to retrieve EVs from typically complex biological samples for further RNA or protein analysis, it is crucial to select an appropriate EV enrichment method for desired applications. The most popular method remains the differential ultracentrifugation (DUC) [5]. However, there are constant needs for a more convenient technique to isolate EVs with better yield and purity. In this study, we describe the use of magnetic beads to enrich EVs as well as EV-miRNAs from plasma samples, an approach that can be readily adopted by laboratories without the need for specialized equipment and yet provides EVs of higher yield and purity that DUC.

A key finding of our study is that EVs in the plasma can be directly captured on magnetic beads and eluted by PBS containing 1% formic acid. Formic acid is a common additive component used to acidify analytes in liquid chromatography. Our NTA data show that overall 57 ± 6% of EVs in the plasma can be recovered in the eluate. Note that the sum of nanoparticles in the eluate and supernatant is only 65% of nanoparticle counts in the plasma. The discrepancy may be due to an EV subset devoid of CD63 expression, incomplete elution and nonspecific adsorption of EVs to glassware and plasticware. The biological activity of eluted EVs remains to be evaluated. In this study, due to currently the lack of universal EV markers, magnetic beads coated with anti-CD63 antibodies were utilized to capture the CD63-positive EV subpopulation. The tetraspanin protein CD63 is one of the classical markers for the majority but not all small EVs (sEV); CD63 may also be found in large EV preparations [14]. Indeed, our NTA data suggest 86 ± 9% of sEV in plasma was recovered using anti-CD63 magnetic beads. In addition, TEM data show the eluate from anti-CD63 magnetic beads contains both small and large EVs (S1 Fig). It will be interesting to study different EV subpopulations by using magnetic beads modified with different capture molecules recognizing cell-specific markers or a combination of tetraspanin molecules such as CD9, CD81, and CD151 [14, 24].

Furthermore, our data show that miRNAs can be extracted directly in plasma using magnetic beads conjugated with complementary nucleic acids. The miRNA extraction efficiency ranges from 87% - 98% as assessed by spiking synthetic miR-126-3p into nuclease-free water and exogenous cel-miR-238-3p into platelet-poor plasma, respectively. (S2 Table) and the concentration of miRNA was estimated according to the respective calibration curve shown in S2 Fig. The miRNA extraction efficiency does not exhibit apparent dependence on the concentration of miRNA (0.1–10 pM) and the medium evaluated. Using this two-step magnetic beads-based (2MBB) approach, EV-associated miRNAs (EV-miRNA) were isolated. Compared to circulating cell-free miRNAs (cf-miRNA), levels of extracted EV-miRNAs appeared more consistent (Fig 4) and better correlated with common CVD protein biomarkers cTnI and NT-proBNP [25, 26] (Fig 5). Improved approaches for the risk stratification and management of cardiovascular diseases (CVDs) are urgently needed to reduce the morbidity and mortality rates. MicroRNAs (miRNAs) are frequently dysregulated and have shown promise as disease biomarkers. Cell-free miRNAs are stably present in human plasma and protected from endogenous RNase activity [27], and the diagnostic and prognostic potential of circulating cf-miRNA has been an active area of research [28, 29]. In particular, both cf-miR-126 and EV-miR-126 have been proposed as a negative regulation biomarker for acute myocardial infarction and chronic CVDs [30–35]. In this work, we further demonstrated that the level of EV-miR-126 was negatively correlated with those of NT-proBNP and cTnI. However, further studies are warranted to validate its clinical significance and specificity.

In addition to the technology advances in miRNA profiling, it may be equally important to study cf-miRNA in specific carriers, as cf-miRNAs may be associated with Argonaut proteins [36, 37], lipoproteins [38], or extracellular vesicles in biological fluids [1, 39, 40]. For instance, miRNAs in epithelial cell adhesion molecule (EpCAM)-positive EVs could only be detected in ovarian cancer patient sera but not in normal controls [41]. Using 2MBB allows us to enrich cell- or disease-specific EVs. In this study, 2MBB was conducted with minimal special equipment required, and an overnight mixing of magnetic beads with the sample is required to attain the desired EV yield. It can be readily integrated with microfluidic systems to provide the precise and automatic control of the process and to shorten the mixing time [21, 42, 43]. The throughput can be further improved by utilizing field-effect transistor (FET) sensors to detect miRNAs directly without PCR amplification [44].

## Conclusions

In summary, we have demonstrated the extraction of EVs and EV-miRNAs in plasma using two sets of magnetic beads sequentially. Immunoaffinity-captured EVs can be eluted for whole-particle assays. Compared to DUC, the yield and purity of recovered EVs are improved by circa 40% and twice, respectively. Our data suggest EV-miR-126-3p may serve as a better biomarker than the total circulating cf-miR-126-3p. Ongoing studies include further clinical evaluations as well as exploring the application of 2MBB in the separation of specific EV subpopulations.

## Supporting information

S1 Fig TEM of particles eluted from anti-CD63 magnetic beads. Transmission electron micrographs of EVs isolated from plasma using anti-CD63 magnetic beads. Eluate contains both small (A) and large EVs (B). Scale bars represent 100 nm.
(TIF)

S2 Fig RT-qPCR calibration curves of synthetic miRNAs. Serial dilutions containing synthetic miR-126-3p and cel-miR-238 molecules were analyzed using RT-qPCR. Results showed that the cycle threshold (*C*_*T*_) values decrease linearly with the increased log concentration of miR-126-3p and (A) cel-miR-238 (B) (*N* = 3).
(TIF)

S1 Table Sequences of relevant target miRNAs detected by the Human Panel A beads. (DOCX)

S2 Table The extraction efficiency of miRNA using magnetic beads. The miRNA extraction efficiency was evaluated by spiking in synthetic miR-126-3p into nuclease-free water and exogenous cel-miR-238-3p into platelet-poor plasma, respectively. The concentration of microRNAs was estimated according to the *C*_*T*_ values and the calibration curves shown in S1 Fig.
(DOCX)
