# Supplementary figures and images for: Two-step magnetic bead-based (2MBB) techniques for immunocapture of extracellular vesicles and quantification of microRNAs for cardiovascular diseases: A pilot study
Source: PLoS One. 2020 Feb 26;15(2):e0229610. doi: 10.1371/journal.pone.0229610 (PMC7043767; doi:10.1371/journal.pone.0229610)

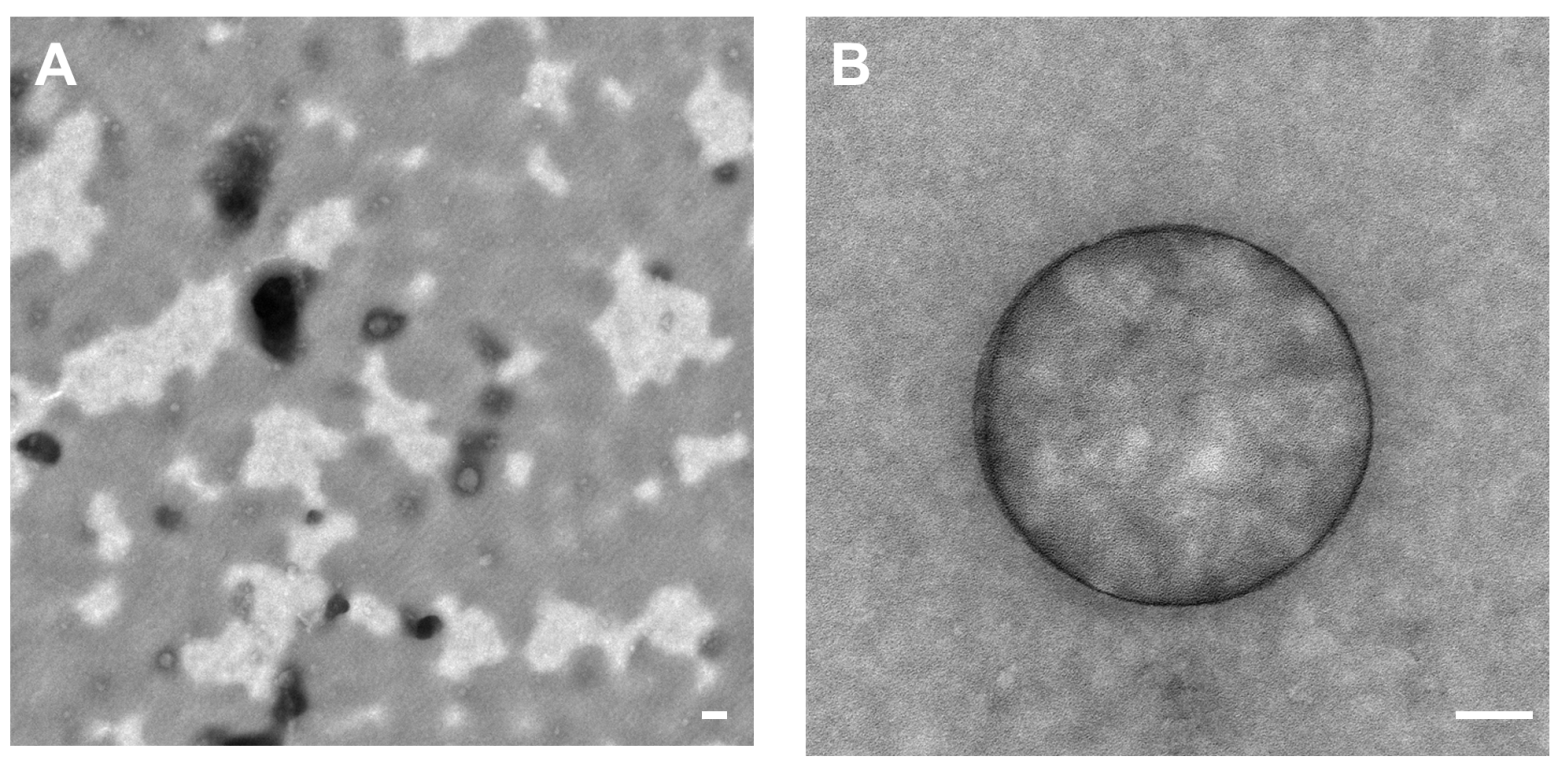

Supplement: S1 Fig — Transmission electron micrographs of EVs isolated from plasma using anti-CD63 magnetic beads. Eluate contains both small (A) and large EVs (B). Scale bars represent 100 nm. (TIF) [file pone.0229610.s001.tif]

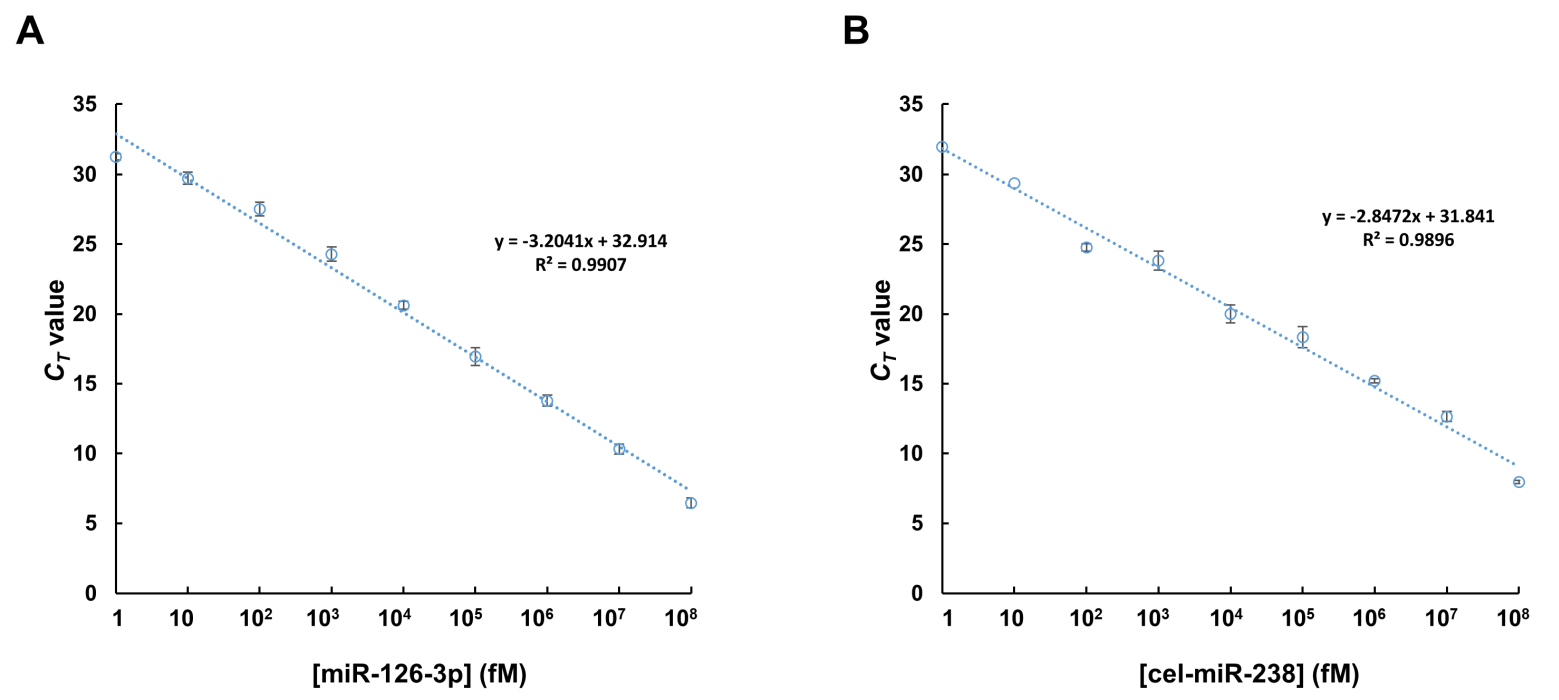

Supplement: S2 Fig — Serial dilutions containing synthetic miR-126-3p and cel-miR-238 molecules were analyzed using RT-qPCR. Results showed that the cycle threshold (CT) values decrease linearly with the increased log concentration of miR-126-3p and (A) cel-miR-238 (B) (N = 3). (TIF) [file pone.0229610.s002.tif]
