# Supplementary material for: Two-step magnetic bead-based (2MBB) techniques for immunocapture of extracellular vesicles and quantification of microRNAs for cardiovascular diseases: A pilot study
Source: PLoS One. 2020 Feb 26;15(2):e0229610. doi: 10.1371/journal.pone.0229610 (PMC7043767; doi:10.1371/journal.pone.0229610)
Supplement: S1 Table — (DOCX) [file pone.0229610.s003.docx]

**S1 Table. Sequences of relevant target miRNAs detected by the Human Panel A beads.**

| **miRbase ID_v18** | **Target Sequence (**5’-3’) |
| --- | --- |
| **cel-miR-238-3p** | UUUGUACUCCGAUGCCAUUCAGA |
| **hsa-miR-21-5p** | UAGCUUAUCAGACUGAUGUUGA |
| **hsa-miR-126-3p** | UCGUACCGUGAGUAAUAAUGCG |
