# Supplementary material for: Two-step magnetic bead-based (2MBB) techniques for immunocapture of extracellular vesicles and quantification of microRNAs for cardiovascular diseases: A pilot study
Source: PLoS One. 2020 Feb 26;15(2):e0229610. doi: 10.1371/journal.pone.0229610 (PMC7043767; doi:10.1371/journal.pone.0229610)
Supplement: S2 Table — The miRNA extraction efficiency was evaluated by spiking in synthetic miR-126-3p into nuclease-free water and exogenous cel-miR-238-3p into platelet-poor plasma, respectively. The concentration of microRNAs was estimated according to the CT values and the calibration curves shown in S1 Fig. (DOCX) [file pone.0229610.s004.docx]

# S2 Table. The extraction efficiency of miRNA using magnetic beads. The miRNA extraction efficiency was evaluated by spiking in synthetic miR-126-3p into nuclease-free water and exogenous cel-miR-238-3p into platelet-poor plasma, respectively. The concentration of microRNAs was estimated according to the *C_T_* values and the calibration curves shown in S1 Fig.

| miRNA | **Medium** | ***C_T_*** | | **[miRNA] (pM)** | | **Efficiency** |
| --- | --- | --- | --- | --- | --- | --- |
|  |  | **Source** | **Extracted** | **Source** | **Extracted** |  |
| **miR-126-3p**^a^ | nuclease- | 23.14 | 23.34 | 1 | 0.87 | 87% |
|  | free water | 19.80 | 19.84 | 10 | 9.79 | 98% |
| **cel-miR-238**^b^ | plasma | 24.46 | 24.58 | 0.1 | 0.091 | 91% |
|  |  | 19.43 | 19.58 | 10 | 8.87 | 89% |

^a^Sequence 5’-ucguaccgugaguaauaaugcg-3’

^b^Sequence 5’-UUUGUACUCCGAUGCCAUUCAGA-3’
